# The Melatonergic System in Mood and Anxiety Disorders and the Role of Agomelatine: Implications for Clinical Practice

**DOI:** 10.3390/ijms140612458

**Published:** 2013-06-13

**Authors:** Domenico De Berardis, Stefano Marini, Michele Fornaro, Venkataramanujam Srinivasan, Felice Iasevoli, Carmine Tomasetti, Alessandro Valchera, Giampaolo Perna, Maria-Antonia Quera-Salva, Giovanni Martinotti, Massimo di Giannantonio

**Affiliations:** 1 National Health Service, Department of Mental Health, Psychiatric Service of Diagnosis and Treatment, Hospital “G. Mazzini”, ASL 4 Teramo, Italy; E-Mail: sfnmarini@gmail.com; 2 Department of Neuroscience and Imaging, Chair of Psychiatry, University “G. D’Annunzio”, Chieti 66013, Italy; E-Mails: giovanni.martinotti@gmail.com (G.M.); digiannantonio@unich.it (M. G.); 3 Department of “Scienze della Formazione”, University of Catania, Catania 95121, Italy; E-Mail: dott.fornaro@gmail.com; 4 Sri Sathya Sai Medical Educational and Research Foundation, Medical Sciences Research Study Center, Prasanthi Nilayam, 40-Kovai Thirunagar Coimbatore, Tamilnadu 641014, India; E-Mail: sainivasan@yahoo.com; 5 Laboratory of Molecular Psychiatry and Psychopharmacotherapeutics, Section of Psychiatry, Department of Neuroscience, University School of Medicine “Federico II”, Naples 80131, Italy; E-Mails: felix_ias@hotmail.com (F.I.); carmine.tomasetti@unina.it (C.T.); 6 Hermanas Hospitalarias, FoRiPsi, Villa S. Giuseppe Hospital, Ascoli Piceno 63100, Italy; E-Mail: alessandrovalchera@gmail.com; 7 Hermanas Hospitalarias, FoRiPsi, Department of Clinical Neurosciences, Villa San Benedetto Menni, Albese con Cassano, Como 22032, Italy; E-Mail: pernagp@tin.it; 8 Department of Psychiatry and Behavioral Sciences, Leonard Miller School of Medicine, University of Miami, 33124 Miami, USA; 9 Department of Psychiatry and Neuropsychology, University of Maastricht, 6200 MD Maastricht, The Netherlands; 10 AP-HP Sleep Unit, Department of Physiology, Raymond Poincaré Hospital, Garches 92380, France; E-Mail: ma.quera@rpc.aphp.fr

**Keywords:** melatonin, melatonergic receptors, serotonin, dopamine, noradrenaline, agomelatine, major depression, anxiety

## Abstract

Melatonin exerts its actions through membrane MT1/MT2 melatonin receptors, which belong to the super family of G-protein-coupled receptors consisting of the typical seven transmembrane domains. MT1 and MT2 receptors are expressed in various tissues of the body either as single ones or together. A growing literature suggests that the melatonergic system may be involved in the pathophysiology of mood and anxiety disorders. In fact, some core symptoms of depression show disturbance of the circadian rhythm in their clinical expression, such as diurnal mood and other symptomatic variation, or are closely linked to circadian system functioning, such as sleep-wake cycle alterations. In addition, alterations have been described in the circadian rhythms of several biological markers in depressed patients. Therefore, there is interest in developing antidepressants that have a chronobiotic effect (*i.e.*, treatment of circadian rhythm disorders). As melatonin produces chronobiotic effects, efforts have been aimed at developing agomelatine, an antidepressant with melatonin agonist activity. The present paper reviews the role of the melatonergic system in the pathophysiology of mood and anxiety disorders and the clinical characteristics of agomelatine. Implications of agomelatine in “real world” clinical practice will be also discussed.

## 1. Anatomy and Physiology of the Brain Melatonergic System

The neurohormone melatonin (*N*-acetyl-5-methoxytryptamine) is prominently, albeit not exclusively, synthesized in the pineal gland and is secreted in a phasic manner, since its circulating levels vary in a daily cycle. Melatonin derives from the precursor tryptophan (taken up from circulating blood) by subsequent steps, implicating tryptophan transformation in serotonin, *N*-acetylserotonin and, finally, in melatonin [1]. Transformation of serotonin in melatonin is regulated by the light-dark cycle, since enzymatic activity of *N*-acetyltransferase (the rate limiting biosynthetic enzyme) is low during daytime or under exposure to light stimuli and higher during dark phases.

Once synthesized, melatonin is released both in the cerebrospinal fluid and in the capillary, which distributes the hormone in multiple body tissues [2]. Melatonin exerts pleiotropic actions on several body compartments and organs; however, these actions are beyond the scope of this review, and the reader is referred to other reviews for more information [3]. Melatonin is regarded as a “chronobiotic” hormone, since it regulates several chronobiological actions and is, in particular, responsible for circadian phase shifting. However, under conditions of total darkness (especially in the Arctic or Antarctic winter, where there is effectively no sunlight for several months), melatonin may still exhibit a diurnal rhythm, albeit that the rhythms become desynchronized. The melatonin-mediated effects are, in turn, under the control of a group of hypothalamic nuclei, mostly the suprachiasmatic nucleus (SCN), which is considered an endogenous circadian pacemaker [4,5]. In seasonal breeders, moreover, melatonin effects are also mediated by the pars tuberalis (PT), which is implicated in photoperiodically-regulated reproduction and the premammillary hypothalamus.

Among the more relevant biological functions exerted by melatonin are the control of the sleep-wake cycle, the modulation of the immune system (including anti-inflammatory properties) and of energy metabolism [6]. Melatonin interacts with at least two receptor types, named MT1 and MT2 [7–11], although adjunctive binding sites have also been described [12,13].

Melatonin receptors belong to the class of G-protein-coupled receptors and are primarily expressed in the CNS; however, they are also widely distributed in other body tissues, together and separately. Within the CNS, the MT1 receptor is prominently expressed in the SCN, the hippocampus, the retina, the caudate putamen, the nucleus accumbens, the substantia nigra and the ventrotegmental area [7,14]. Notably, most of these areas belong to the central dopaminergic pathways, suggesting a tight correlation between the melatonergic and monoaminergic systems, at least the dopaminergic one. MT1 receptors are also found in several other hypothalamic nuclei and brain areas, such as the paraventricular nucleus, the periventricular nucleus, the supraoptic nucleus, the diagonal band of Broca, the Meynert nucleus, the tuberomammillary nucleus and the mammillary bodies [14]. The MT2 receptor has been mostly found in the hippocampus, the SCN and the retina [7]. Outside the CNS, MT1 receptors are thoroughly distributed in several tissues, while expression of MT2 receptors is more restricted [7]. Expression of both MT1 and MT2 receptors has also been reported in neurons and glial cells of the cortex, thalamus and cerebellar cortex. Moreover, both receptors are expressed in the pineal gland [15], which is consistent with reported melatonin autocrine and paracrine actions.

Intriguingly, expression of melatonin receptors in both central and peripheral tissues is affected on a circadian basis, since mRNA expression of the MT1 receptor has been found to be increased in rodents during daytime [16]. Binding to the MT1 receptor was also found to be increased during the daytime and by light exposure during the nighttime, while MT1 mRNA expression was reduced by this latter procedure [15]. Despite an increase in mRNA expression during the daytime, however, surface expression of the melatonergic receptor in SCN neurons has been reported to be very low during the day and high at night, paralleling melatonin’s trough and peak [17]. These findings suggest that the levels of circulating melatonin elicit a feedback regulation on the surface receptor amount, causing a downregulation of receptors with blood peaks. At the same time, surface receptors are more expressed when melatonin levels are expected to be higher and *vice versa*. This group of feedback regulations allows the system to preserve its homeostasis and to provide fine-regulation with external light-dark stimuli. However, regulation of melatonin receptor expression is under multiple other biological factors, e.g., estradiol levels, which cooperate to modulate melatonergic signaling in its different steps.

MT1 receptor signaling occurs mainly through interaction with inhibitory G-proteins and subsequent reduction of intracellular cAMP levels, a decrease in protein kinase A activity and reduced phosphorylation of the transcription factor, CREB [18]. However, in an artificial system, *i.e*., cultured cells, melatonin has also been demonstrated to increase cAMP levels via interaction with the MT1 receptor [19], although the actual relevance of these *in vivo* observations remains questionable.

MT2 receptors couple to multiple and diverse transduction pathways, depending on the biological system taken into consideration and ranging from inhibition of cAMP synthesis to the increase of protein kinase C activity or from inhibition of guanylyl cyclase signaling to increase cGMP levels [20–22].

At the subcellular level, melatonin modulates the activity of a number of ion channels and affects intracellular ion levels [23,24]. The hypothalamic SCN and the hippocampus are two major sites of melatonin action in the CNS. The SCN activity is inhibited by melatonin via MT1 receptors [25], mostly during the daytime, when the SCN neuronal activity is higher. However, melatonergic inhibition of SCN activity is blunted by melatonin itself through the regulation of surface receptor expression [26] by their desensitization. Desensitization occurs after exposure to melatonin by a beta-arrestin-1-dependent mechanism [27], requiring a phosphorylation on the MT1 and MT2 receptor *C*-terminal [28]. Melatonergic receptor desensitization is mainly associated to receptor downregulation. Prevention of melatonergic receptor desensitization has been obtained by depolymerizing microtubules and the blockade of receptor internalization [29]. However, in SCN cells, exposure to physiological concentrations of melatonin caused the reversible desensitization and downregulation of MT2 receptors and the desensitization of MT1 receptors, however, without their downregulation [30]. Therefore, multiple molecular mechanisms may concur to diminish melatonin-mediated signaling.

In the hippocampus, melatonin has been reported to increase the firing rate of CA1 neurons through activation of MT2 receptors [30]. In hippocampal slices, melatonin disrupted long-term potentiation in a dose-dependent fashion [30]. Both effects were prevented by application of a MT2 receptor antagonist and were lacking in MT2 gene knock-out mice, but not in MT1 defective mice [31]. Melatonergic signaling via the MT2 receptor in hippocampus has been described to substantially affect cognitive behavior in preclinical paradigms. Knock-out mice for the MT2 gene were found to perform worse than wild-type littermates at the elevated plus-maze behavioral task, a procedure investigating learning and memory abilities [32], possibly implying an impairment in hippocampal-mediated synaptic plasticity. It has also been demonstrated that agomelatine, a potent melatonin receptor agonist drug that strongly binds to and stimulates the activity of melatonin MT1 and MT2 receptors, showed cognitive enhancing properties, at least in preclinical studies [33,34].

The physiology of melatonergic signaling has been elucidated only in part. In mammals, melatonin exerts a main action on phase shifting, which reflects a feedback loop between the pineal gland and the endogenous circadian pacemaker, *i.e.*, the SCN [3]. Within this feedback loop, melatonin controls the amplitude and phase of circadian oscillation. Namely, the hormone exerts phase shifting via MT2 receptors and affects neuronal firing by MT1 receptors [35]. In functional agreement with these actions, melatonin also favors sleep initiation by a number of mechanisms, including MT1 receptor-mediated effects on the hypothalamic sleep switch [36] and modulation of discrete thalamic projections to the cortex that are implicated in sleep stage transitions [37]. Moreover, melatonin also takes part in sedating and anxiolytic effects.

Recently, Ochoa-Sanchez *et al.* [38] demonstrated that the melatonin MT2 receptor was involved in the regulation of non-REM (NREM) slow wave sleep (SWS), and the MT2 selective agonist, UCM765, increased NREM by activating the reticular thalamus neurons, where the MT2 receptors are located. The melatonin MT2 receptor also mediates anxiety function [39]. On the other hand, it has been demonstrated that the melatonin MT1 receptor is mostly involved in the control of REM sleep [40].

## 2. Interactions between Melatonergic System and Monoaminergic Systems

The main role of the pineal gland is to produce melatonin in response to the absence of light stimuli, which may, in turn, activate a glutamate-mediated response from retinal receptors to SCN GABAergic neurons, thereby generating an environment-to-endocrine input translation that is at the basis of circadian rhythms in humans [41]. Located in the middle of the brain, although externally to the blood-brain barrier, the pineal gland represents a powerful triage organ, where neurotransmission signals from the SCN are converted to endocrine secretion, which, in turn, may regulate other monoaminergic neurotransmitter systems, such as dopamine, norepinephrine and serotonin. For instance, recent reports demonstrated that chronic melatonin treatment in animal models of aging may help normalize levels of all monoamines, such as dopamine, serotonin and norepinephrine, thus contrasting age-related impairment in catecholamines neurotransmission [42].

Here, we review the complex interconnections between catecholaminergic systems and melatonin neuroendocrine secretion.

### 2.1. Serotonin Is the Main Controller of Circadian Clocks

Melatonin secretion is obviously tightly dependent on the availability of serotonin in pinealocytes. Since serotonin is the precursor of melatonin, this neurotransmitter is, indeed, the principal actor of the light/dark circadian regulation of melatonin secretion [43]. The SCN contains a hyper-dense serotonergic terminal plex [44]. Early studies demonstrated that serotonin administration or serotonergic agonist agents may phase-shift the circadian SCN pacemaker [45]. Moreover, lesions in the raphe nuclei may cause a decrease in circadian amplitude or a phase change [43]. Several studies demonstrated multiple mechanisms of modulation by serotonin receptors on the circadian clock. Serotonin, indeed, may postsynaptically increase potassium currents in a subset of SCN neurons, in order to alter circadian phases [46]. Moreover, SCN-evoked currents may be presynaptically inhibited by serotonin, through a direct reduction in excitatory stimuli originating from the retinal tract [42]. However, more recent reports demonstrated a more complex regulation of SCN rhythms by serotonin. Indeed, serotonin agonists are able to phase-shift circadian clocks only when serotonin release has been previously decreased or in *in vitro* environments devoid of serotonin concentrations. When serotonergic agents are pre-applied to SCN neurons, a new application of serotonin is less able to phase shift the circadian clock, thereby demonstrating that the SCN is affected by serotonin only, depending on the pre-existent serotonergic signaling [47]. Substantially, serotonin may act as a synchronizer with the same effects as light on SCN neurons [48]. Indeed, synchronization of circadian clocks by both light and serotonin coincide with modulation of specific clock genes, *Per1* and *Per2* [49–51]. However, synchronizing effects of serotonin on circadian rhythms have been demonstrated to occur only at daytime. When administered at nighttime, serotonin may only modulate light-induced phase-shifts in clock genes, but is not able to phase-shift clocks by itself [52]. Recent studies demonstrated that the reasons for this “paradox” reside in the light/dark-dependent differential expression of serotonergic-specific receptors in the SCN [53].

Emerging evidence supports the view that melatonin may also regulate serotonin secretion in a retrograde circadian feedback loop. Serotonin, indeed, is also secreted by raphe nuclei with a clear downward trend during the night and an upward increase in the daytime [54]. Melatonin, on the other hand, may inhibit raphe neuron firing, possibly through acting on MT1 receptors [55].

### 2.2. Norepinephrine Controls Limiting Steps of Enzymatic Melatonin Production

Several studies demonstrated that melatonin secretion is not tightly dependent upon light, since in complete darkness, melatonin is produced by SCN-triggered stimulus. Circadian rhythms are synchronized by light-dark cycles.

The limiting enzymatic step of melatonin production in pinealocytes is the *N*-acetylation of serotonin by aryl-alkylamine-*N*-acetyl-transferase (AANAT), which has been demonstrated to increase its functioning levels at night [56]. In fact, it is possible to assume that AANAT functioning is the real circadian clock at the pineal gland level.

Several studies have demonstrated that the nocturnal increase in melatonin secretion is directly related to norepinephrine nightly release by SCN to pineal gland. Norepinephrine, indeed, may stimulate alpha- and beta-adrenergic pineal receptors, which, in turn, trigger a cAMP-dependent increase in PKA intracellular activity that induces the CREB-mediated transcription of AANAT protein [57]. This cascade provides a rapid increase in AANAT activity, which results in melatonin formation in a 2 h time range after norepinephrine stimulus. However, other norepinephrine-related mechanisms have been implicated in melatonin secretion control. Indeed MAPK activation by adrenergic receptors has also been involved in AANAT pinealocytes responses [58]. Moreover, adrenergic signaling has been demonstrated to modulate histone deacetylation and phosphorylation by means of a MAPK-dependent mechanism, which suggests a further control on AANAT functioning (for a review, see [59]).

Emerging evidence suggests that norepinephrine control of melatonin secretion may be under a dynamic presynaptic control. Indeed, early studies demonstrated that, besides SCN innervation, the pineal gland receives projections from sphenopalatine, otic and trigeminal ganglia [60], which have been reported to secrete substance P [61]. Recent works demonstrated that pinealocytes are enriched with substance P receptors [62] and that substance P may inhibit norepinephrine-induced AANAT activation and melatonin secretion, though it does not impair basal levels of AANAT functioning [63].

As a further suggestion of presynaptic control of norepinephrine-mediated melatonin activity, Koch and coworkers recently demonstrated that phytocannabinoids (e.g., tetrahydrocannabinol) application to rat pineal gland cultures may reduce stimulation of melatonin secretion by norepinephrine [64]. Further studies by the same group recognized endocannabinoid receptors and metabolizing enzymes in pinealocytes, thereby demonstrating a possible endocannabinoid-dependent control of norepinephrine-stimulated melatonin secretion [65].

Conversely, some hormones, such as insulin, may enhance norepinephrine-mediated melatonin synthesis and AANAT activity [66].

### 2.3. Melatonin-Dopamine Reciprocal Interactions: Molecular Bases for Neuropsychiatric Disorder Pathophysiology

Emerging evidence supports the bi-univocal relation between melatonergic and dopaminergic transmission. Dopamine, indeed, is present in sympathetic nerves projecting to the pineal gland, not only as a precursor of norepinephrine, but also as a neurotransmitter, which has been demonstrated to have a crucial role in melatonin secretion control. Indeed, pinealocytes express high levels of dopamine D4 receptors (D4R), whose expression has the singular ability of being dynamically regulated in the pineal gland based on night/day circadian retinal synchronization [67]. Recent evidence demonstrated that D4R expression is directly controlled by norepinephrine action at alpha1- and beta1-adrenergic receptors, since beta-adrenergic agonists may increase D4Rs on the pinealocyte cell surface [68]. Notably, D4R expression in pinealocytes seems to be controlled by a “double gate” mechanism, which requires the thyroid hormone-mediated activation of II iodothyronine deiodinase (Dio2), which transforms T4 into T3 active hormone, which acts simultaneously on the adrenergic-mediated cAMP formation at the basis of D4R translation [67].

Therefore, dopamine seems to exert a complex modulatory control on melatonin synthesis, highly dependent on light/dark cycles. Recent evidence demonstrated an entangled mechanism by which dopamine may regulate norepinephrine-dependent melatonin secretion. D4R, indeed, may form heteromeric complexes with both alpha1- and beta1-adrenergic receptors. These heteromers, whose formation is controlled by light–dark circadian rhythms, may enable D4R to modulate the adrenergic-mediated activation of MAPK and Akt cascades, which, in turn, promote melatonin synthesis and secretion [69].

On the other hand, melatonin has been demonstrated to control dopamine signaling in selected regions of the forebrain. Indeed, melatonin may inhibit dopamine release in hypothalamic areas (tuberoinfundibular region), as well as in ventral hippocampus [70]. The decrease in dopamine concentrations in these areas occurs simultaneously with a concurrent increasing in tyrosine hydroxylase activity [71]. Melatonin seems also able to reduce cortical glutamate-mediated stimulation of striatal responses [72]. These effects may be directly dependent on the reduction in dopamine striatal release by nigrostriatal fibers induced by melatonin. Moreover, it has been demonstrated that melatonin may directly reduce NMDA glutamate receptor functions in striatal neurons, thereby reducing NMDA-mediated long-term synaptic responses in this brain region [73]. Other studies demonstrated that melatonin may directly act on dopaminergic receptor functions in the brain. Indeed, melatonin has been reported to increase dopamine D2 receptors (D2R) affinity in rat striatum [74]. In contrast, melatonin may dose-dependently counteract the dopamine D1 receptors (D1R)-mediated cAMP enhancement in neurons [75].

Melatonergic receptors (MT1, MT2) are widely distributed in the brain, above all, in hippocampus, cortex, hypothalamus and cerebellum [76–78]. Moreover, melatonin receptors seem to have special relations with dopaminergic systems. Indeed, MT1 receptors are localized in ventral tegmental area and striatum (above all, nucleus accumbens shell). Surprisingly, these receptors have light/dark-dependent expression, with high levels during the night and low levels during the day [14]. This specific connection between melatonin receptors and dopaminergic sites may have an essential role in the pathophysiology of behavioral disorders that depends upon dopaminergic dysfunctions.

Indeed, it has been demonstrated that cocaine reward sensitization yields a critical dependence on daylight, with pineal gland having a crucial role in this diurnal reward [79]. Moreover, pharmacological doses of melatonin may block the cocaine-induced diurnal behavioral sensitization [80]. Inversely, cocaine protracted administration may reduce MT1 receptor expression in striatum [81].

Last, melatonin has been demonstrated to have protective effects in animal models of dopaminergic dysfunctions, such as 6-hydroxydopamine-induced parkinsonisms [82] or rotenone-induced motor disturbances [83].

Recently, a significant association of the MT1 receptor haplotype ATG has been reported in antipsychotic-treated schizophrenic patients that do not develop tardive dyskinesia after prolonged treatments [84].

## 3. Circadian Disturbances in Depression

The complex relationships between the endogenous circadian pacemaker and the development of depressive symptoms are far from being elucidated [85]. The worsening of diurnal mood variation (DMV) with the early morning is a classic symptom of the melancholic features of major depressive disorder (MDD) and is one of the time-linked symptoms that has promoted speculation about the role of the circadian system in its pathogenesis [86]. MDD seems to be related to a disruption in the central circadian clock function and not to an alteration in a specific rhythm [85].

In addition, the type of rhythm abnormality seems to be highly variable in depressed patients, including phase advance or phase delay of rhythms and increase or decrease in the rhythm amplitude [87]. There is substantial evidence that circadian rhythms are more attenuated in MDD than euthymic states, with decreased circadian amplitudes in core body temperature, motor activity, thyroid-stimulating hormone, norepinephrine (NE) and cortisol, as was found in several studies [88]. These decreased amplitudes might result from the weakened output of the endogenous oscillator and are one of the most relevant chronobiological abnormalities in depression that may be corrected by antidepressant drugs [87,88]. A phase advance of the rhythm of cortisol, adrenocorticotropin, prolactin and growth hormone has also been noted in depressed patients [85].

## 4. Chronobiotic Properties of Agomelatine

Agomelatine (Valdoxan^®^/Thymanax^®^) (S20098, *N*-[2-(7-methoxynaphth-1-yl)ethyl]acetamide) was first reported in the literature in 1992, among a series of synthetic naphthalene melatonin analogs. Various animal models of abrupt shifts and disorganization of the light-dark cycle, of free-running conditions, as well as of delayed sleep-phase syndrome have shown that agomelatine accelerates the resynchronization of circadian rhythms of locomotor activity and relevant biological parameters (*i.e.*, body temperature, secretions of hormones) [33]. The resynchronizing activity of agomelatine has been mainly shown in both nocturnal (rats, mice, hamsters) and diurnal (Arvicanthis mordax) animals. The ability of agomelatine to synchronize rest-activity rhythms in free-running animals requires the integrity of the SCN [34].

The accelerating effect of agomelatine was particularly notable if treatment was started three weeks prior to the induced phase shift [89,90]. Agomelatine treatment did not cause any major change in corticosterone or adrenocorticotropic hormone concentrations, vasopressin, corticotropin-releasing hormone and mineralocorticoid receptor mRNAs levels, which suggests that the mechanism of agomelatine action is not related to hypothalamic-pituitary adrenocortical axis changes. This study showed that agomelatine displays some characteristics of antidepressant drug action in the transgenic mouse model, effects that could be partially related to its chronobiotic properties [85].

## 5. Pharmacodynamics and Pharmacokinetics of Agomelatine

**Figure 1:**
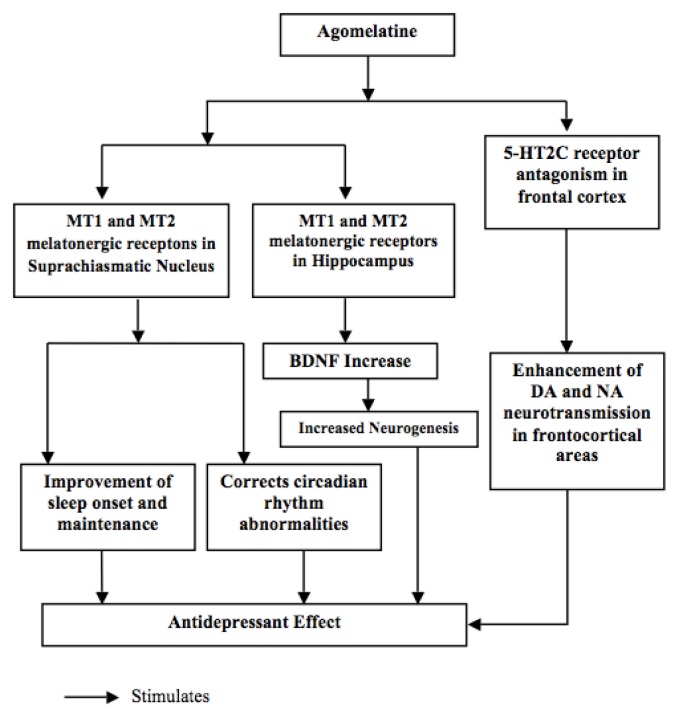
Mechanism of action of agomelatine (adapted from Srinivasan *et al.* [105]).

Agomelatine shows agonistic activity with high affinity for melatonin MT1 and MT2 receptors and an antagonist activity with moderate affinity for 5HT2C [91] (Figure 1). However, even if there is no question from the animal data that 5HT2C antagonism occurs, such antagonism in humans has been questioned [92,93]. 5HT1A and 5HT2B receptors are not thought to be responsible for agomelatine clinical effects, due to the low affinity of the drug for these receptors [94]. No significant affinity for any of the monoamine transporters or for adrenergic, noradrenergic, dopaminergic, muscarinic, histaminic and benzodiazepine receptors has been reported [95]. The binding affinity of agomelatine for MT1 and MT2 is similar to melatonin. The literature reported that antidepressant efficacy could be related to melatonin secretion through monoaminergic mechanisms [96,97], even if controversial data regarding blood melatonin concentrations in MDD were reported [98–104].

Moreover, in experiments conducted on animals, agomelatine has demonstrated the ability to increase adult hippocampal and prefrontal cortex neurogenesis, to enhance expression of brain-derived neurotrophic factor (BDNF) and to trigger several cellular signals, *i.e.*, protein kinase B (Akt), extracellular signal-regulated kinase 1/2 (ERK1/2) and glycogen synthase kinase 3β (GSK3β) [106]. It has also been reported that agomelatine may have beneficial effects on hippocampal neurogenesis in the stress-compromised brain of rats [107]. Most pre-clinical models suggested that either the 5HT2C antagonist effect or the MT1/MT2 agonist effects alone are not sufficient to account for the antidepressant properties of the drug, at least in predictive animal models [91–93]. Tardito *et al.* [108] proposed that the molecular-cellular effects of agomelatine and, therefore, its antidepressant activity, may be the result of a synergistic action between its agonism at MT1/MT2 and antagonism at 5-HT(2C) receptors. The antidepressant properties of agomelatine, related to its effect on neurogenesis, cell survival, BDNF, activity-regulated cytoskeleton associated protein (Arc) and stress-induced glutamate release, are due to this synergistic action. Intriguingly, agomelatine is the only one able to resynchronize these effectors at distinct levels, circuital and intracellular [102].

After oral administration, agomelatine is rapidly (Tmax ranging from 0.5 to 4 h) and well absorbed (80%), but its bioavailability is relatively low (<5% at the therapeutic oral dose) due to its high first-pass metabolism [109,110], which may be of concern, especially in elderly patients or in subjects with liver disorders. In humans, agomelatine has a moderate volume of distribution of approximately 35 L, a plasma protein binding of 90%–94% (albumin and alpha l-acid glycoprotein) and a short plasma half-life (1–2 h) [111]. At therapeutic levels, agomelatine blood concentration increases proportionally with dose; at higher doses, a saturation of the first-pass effect may occur. The bioavailability is 2-fold higher for women compared to men [97]. About 90% of agomelatine is metabolized by cytochrome P450 (CYP) 1A2 (hydroxylation) and about 10% by CYP 450 2C9 (demethylation) isoforms. At higher serum concentrations, also CYP 450 2C19 is involved in metabolism. Metabolites are conjugated with glucuronic acid and then sulfonated. About 80% of the drug is eliminated through urinary excretion of the metabolites (61%–81% of the dose in humans), whereas a small amount of the metabolites undergoes fecal excretion [112].

## 6. Agomelatine in the Treatment of Major Depressive Disorder

Major depressive disorder is one of the most disabling and common psychiatric disorders. Recent data estimate a lifetime prevalence of MDD at 16.6% and the one-year prevalence at 6.7% [113–116]. MDD is a leading cause of premature death and ongoing disability [117,118]. Psychopharmacological treatments include a number of antidepressant drugs; however, over 60% of treated patients respond unsatisfactorily, and almost 20% of patients become refractory to the treatments [119–121]. Patients who respond satisfactorily to the treatments benefit from reduced suicide rates, increased participation in the workforce, reduced secondary alcohol or other substance misuse and decreased risk of cardiovascular disease [122,123]. In clinical studies, patients with a reduction of 50% or more on the Hamilton Depression Rating Scale (HAMD) total score at endpoint are considered responders to treatment; remission, which represents complete or near complete symptom resolution, including resolution of functional impairment, is commonly defined as an HAM-D total score of ≤7 [118].

### 6.1. Materials and Methods of Literature Review

Searches of the Medline database from 1988 through August 2012 and the PsycInfo/Embase database from 1988 through January 2013 were carried out with the help of a professional librarian (M.C.), restricted to the English language. The search term “agomelatine” was combined with “depression”, “major depression”, “major depressive disorder”, “mood disorders”, “placebo”, “efficacy” and “adverse effects” to identify relevant original research and review articles. All citations were screened, and the full texts of peer-reviewed journal articles considered relevant to the purposes of the review were obtained. Furthermore, articles in press were included, if relevant. Bibliographies were scanned to locate additional relevant publications, even those older than 1988.

### 6.2. Acute Phase Trials with Agomelatine *versus* Placebo

There are eight acute phase trial studies (five published and three unpublished) comparing agomelatine *versus* placebo (see Table 1). From published studies, three trials showed that agomelatine was more effective than placebo on the total HAMD score [124–126]; one trial reported that only 50 mg of agomelatine provided a statistically significant improvement in the HAMD score from first baseline visit through week 8, whereas 25 mg of agomelatine did not [101]; one trial reported that 25 mg of agomelatine was more efficacious based on the HAMD total score compared to placebo throughout the treatment period, whereas 50 mg of agomelatine did not [102].

From unpublished studies, one unpublished trial reported no significant differences in HAMD and Clinical Global Impression scale (CGI) scores in agomelatine *versus* placebo compared to fluoxetine *versus* placebo groups. (CL3-022) [97]; two studies (CL3-023 and CL3-024) were failed trials.

### 6.3. Antidepressant Efficacy in Active Comparator Trials

Agomelatine treatment efficacy, based on HAMD, CGI and the Montgomery-Asberg Depression Scale (MADRS), have been rated by several studies. Treatment with agomelatine systematically showed, at least, comparable efficacy with other antidepressants. Numerous studies have been conducted comparing agomelatine and venlafaxine. It is interesting that although antidepressant efficacy on HAMD was similar, CGI improvement was significantly higher and statistically significant for agomelatine compared to venlafaxine [127,128]. Based on MADRS scores at the end point for response and remission rates, antidepressant efficacy was similar in the two treatment groups [129]. After six weeks of treatment, the HAMD final score, as well as CGI was significantly better for agomelatine than for sertraline [130]. Over eight weeks, the mean decrease in HAMD total score was significantly greater with agomelatine than fluoxetine [131]. Based on HAMD scores, agomelatine was reported to be statistically non-inferior to escitalopram at six weeks [132].

One study compared the efficacy of agomelatine and sertraline in the treatment of depression and anxiety in depressed patients with type 2 diabetes mellitus [133]. Agomelatine was effective in the treatment of depression and anxiety, as well as in the improvement of health-related behaviors, in depressed patients with non-optimally controlled type 2 DM.

### 6.4. Anhedonia in Major Depressive Disorder

Anhedonia is defined as a loss of interest and lack of reactivity to pleasurable stimuli. It is considered a core symptom of MDD, a predictor of poor outcome [134], a common residual symptom after treatment [135] and associated with dysfunctions within the brain reward system [136,137]. In the first study where agomelatine was reported to be effective in the treatment of anhedonia, Di Giannantonio *et al.* found a significant improvement in the Snaith Hamilton Rating Scale (SHAPS) [138]. Moreover, after eight weeks of treatment, agomelatine showed a more relevant reduction compared to venlafaxine in SHAPS score [124].

### 6.5. Sleep in Major Depressive Disorder

Sleep and daytime functioning are important aspects of major depressive disorder. Agomelatine showed an important difference in getting to sleep and quality of sleep in comparison with venlafaxine and sertraline [105]. Agomelatine was superior to venlafaxine, but no statistically significant difference was found when compared to sertraline in ease of awakening and integrity of behavior after awakening [117,120].

Two open-label studies evaluated agomelatine efficacy on sleep parameters in patients with MDD [139,140]. No change in rapid eye movement (REM) latency, amount of REM or REM density was observed. Agomelatine improved sleep continuity and quality, and it increased sleep efficiency, time awake after sleep onset and the total amount of SWS [128]. Agomelatine treatment improved very early NREM and REM sleep [129].

Recently, agomelatine has been evaluated on nighttime sleep and daytime condition compared to escitalopram [122]. Agomelatine was associated with reduction in sleep latency, preserved the number of sleep cycles and reduced daytime sleepiness. In a recent open-label study, 80% of patients with MDD receiving a flexible dose of agomelatine showed significant improvements at all visits in Leeds Sleep Evaluation Questionnaire (LSEQ) [127].

### 6.6. Sexual Function

An important and non-negligible side effect of antidepressants is represented by sexual dysfunction. Kennedy *et al.* [118] found that treatment related sexual dysfunctions were significantly lower in the agomelatine group, whereas venlafaxine was associated with greater deterioration in the domains of desire and orgasm of the Sex Effects Questionnaire. In a randomized, placebo-controlled, eight-week study involving healthy male volunteers, agomelatine was shown to have better sexual acceptability than paroxetine. In fact, the Psychotropic Related Sexual Dysfunction Questionnaire (PRSEXDQ), reported better scores for agomelatine, similar to placebo, compared to paroxetine [141].

### 6.7. Anxiety Symptoms within Depression

Anxiety symptoms are common in patients with MDD. Some trial studies evaluating agomelatine treatment efficacy in depressed patients reported Hamilton Anxiety (HAMA) scale scores. Final HAMA scores were similar when agomelatine was compared to paroxetine, fluoxetine and venlafaxine [114,117,118,121]. Agomelatine was superior in reducing HAMA scores compared to sertraline [120].

### 6.8. Discontinuation Symptoms

Discontinuation symptoms in MDD have been evaluated in only one randomized, double-blind, placebo controlled study, with paroxetine as the active comparator [142]. No significant differences were present in the Discontinuation-Emergent Signs and Symptoms scale (DESS) between patients who stopped or continued agomelatine treatment. On the other hand, DESS scores were higher in patients discontinuing paroxetine, who reported insomnia, dreaming, dizziness, muscle ache, nausea, diarrhea, rhinorrhea and chills. These data suggest that agomelatine is not associated with discontinuation symptoms. In an active comparator trial, discontinuation rates were fewer for agomelatine than venlafaxine, sertraline and fluoxetine [119–121], but similar to paroxetine [114].

### 6.9. Responders, Remitters and Relapse Prevention

Remission is the final goal of antidepressant treatment. Six studies reported response and remission rates (three after six months of treatment and three in the acute phase). At six months, the efficacy of agomelatine was superior to venlafaxine in CGI scores [117], but no significant differences in the proportion of responders and remitters were found [117,119]. The responders’ proportion was superior for agomelatine compared to sertraline by HAMD, but no differences were found in the proportion of responders by CGI or remitters by HAMD or CGI [120]. Compared to placebo, in acute phase trials, response rates were significantly higher for agomelatine [114–116]. Only one study reported higher levels of remission rates for agomelatine after an eight-week treatment period [114]. Sparshatt *et al.* conducted a multicenter naturalistic evaluation of the use of agomelatine over a two-year period, in order to provide a picture of its clinical value in the treatment of depression [143]. Agomelatine was largely used in difficult-to-treat or refractory patients. After 12 weeks of treatment, a substantial number of patients improved by at least one point of the CGI (severity) scale.

Two trial studies (one unpublished and one published) investigated long-term antidepressant effect of agomelatine treatment compared to placebo, regarding relapse prevention. The incidence of relapse over six months was significantly lower with agomelatine [144]. No significant differences in relapse rates were shown in the unpublished study (CL3-021) [97].

### 6.10. Serum Transaminases

Servier Laboratories reported that agomelatine may cause a dose-related elevated liver function test (LFT), specifically serum transaminases >3-times the upper limit of normal [145]. The European Medicines Agency requires the monitoring of liver function during treatment at all doses [97].

Two studies reported notable aminotransferase elevations in 2.4% and 4.5% of patients in treatment with agomelatine at 50 mg, but not with agomelatine at 25 mg or with placebo [146,147]. These LFT increases were isolated, mainly within the first month of treatment, and no clinical signs of liver damage were found. A higher proportion of patients with LFT elevations had a history of cholecystitis, gallbladder disorder or hepatic steatosis. For these reasons, agomelatine is contraindicated in patients with hepatic impairment. Consequently, it is a condition of treatment that LFTs should be performed for all patients at the initiation of treatment, then periodically after around six, 12 and 24 weeks and, thereafter, when clinically indicated [148]. If an increase in serum transaminases occurs, blood liver function analyses must to be repeated within 48 h, and it is necessary to discontinue the therapy if such an increase is three-times the upper limit of the normal range. The liver function test must be evaluated until serum transaminases return to the normal range.

### 6.11. Limitations of Agomelatine Trials in Major Depressive Disorder

Despite the majority of positive study results regarding agomelatine in the treatment of MDD, limitations of the reviewed studies should be considered. For example, inclusion and exclusion criteria employed in the trials may have somewhat favored individuals who would respond to treatment. In fact, patients with a recent history of suicidality, electroconvulsive therapy, psychotic features or recent substance abuse were excluded in almost all studies. Moreover, most of the studies with an active comparator arm employed relatively low dosages of venlafaxine, paroxetine, sertraline and fluoxetine, which may have improved the relative efficacy of agomelatine [149]. Four trials with an active comparator arm did not have a placebo group [117,118,140,141]. These studies reported high rates of response, but the lack of placebo groups makes it difficult to place the high rates of response in a proper context. In addition to high response rates, two studies reported that the differences in antidepressant efficacy were not statistically significant when comparing agomelatine with venlafaxine or paroxetine [117,119]. In both of the studies, antidepressant efficacy was only a secondary outcome, and no clear description of statistical power was provided. Thus, the similar rates of antidepressant efficacy between agomelatine and the comparator agents may have been affected by the relatively low dose of venlafaxine (75–150 mg/day) and paroxetine (20 mg/day) and by an inadequate power when comparing antidepressant efficacy between these agents.

However, despite these shortcomings, the placebo-controlled trials reported improvements in depression rating scale scores (*i.e.*, 2–3 points) that were similar to responses reported to the Food and Drug Administration involving a number of agents approved for the treatment of MDD [139]. In 2006, agomelatine was denied marketing authorization in Europe, due to a reported lack of efficacy [148]. Since that time, additional studies demonstrating agomelatine’s efficacy have been published, and in November 2008, the committee for medicinal products for human use of the European Medicines Agency provided marketing authorization for treating MDD episodes in adults with agomelatine [120].

## 7. Conclusions

MDD is one of the most disabling and common psychiatric disorders. It should be noted that some unpublished studies reported no significant differences in HAMD and CGI scores between agomelatine *vs.* placebo compared to fluoxetine/paroxetine *vs.* placebo groups, but no significant differences in relapse rates were also shown when agomelatine was compared to placebo. On the other hand, published data reported agomelatine efficacy, compared to placebo and based on HAMD, in the treatment of major depressive disorder. Howland *et al.* [150,151] have suggested that a publication bias may be present, as the favorable studies have generally been published and unfavorable studies have generally not been published. When compared to other antidepressants (venlafaxine, sertraline, fluoxetine and escitalopram), agomelatine showed, at least, comparable efficacy.

The efficacy of agomelatine on the dimension of anhedonia may be of particular importance in the treatment of MDD with anhedonic features. In fact, on the basis of SHAPS scores, agomelatine was reported to be effective in the treatment of anhedonia. In particular, agomelatine showed a more relevant reduction in SHAPS scores when compared to venlafaxine after eight weeks of treatment. Some studies reported that agomelatine was similar to sertraline and superior to venlafaxine and escitalopram in the improvement of sleep parameters in patients with MDD. Lower deterioration in the domains of desire and orgasm of the Sex Effects Questionnaire were reported when agomelatine was compared to venlafaxine. In healthy male volunteers, agomelatine was shown to have better sexual acceptability than paroxetine.

Discontinuation rates for any cause were fewer for agomelatine than venlafaxine, sertraline and fluoxetine. Moreover, data suggest that agomelatine is not associated with discontinuation symptoms, even if this potential side effect warrants further investigation, especially regarding long-term risks. About responder and remission rates, data are contrasting, even if agomelatine was largely used in difficult-to-treat or refractory patients. The incidence of relapse over six months was significantly lower with agomelatine.

## Figures and Tables

**Table 1:** Published placebo-controlled and/or active comparator studies of agomelatine in the treatment of major depression. HAMD, Hamilton Depression Rating Scale.

Authors	Reference	Study Design	Comparator/Active Control	Number of Patients	Duration	Agomelatine Dosage (mg/day)	Results
Loo *et al.*, 2002	123	Placebo-controlled dose range study	Placebo/Paroxetine 20 mg/day	711	8 weeks	1, 5 and 25	Agomelatine at 25 mg was statistically more effective than placebo
Montgomery *et al.*, 2004	142	Randomized, double-blind, placebo-controlled discontinuation study	Placebo/Paroxetine 20 mg/day	335	12 weeks	25	Agomelatine was effective and had less potential to cause discontinuation symptoms than paroxetine
Kennedy & Emsley, 2006	124	Randomized, double-blind, placebo-controlled study	Placebo	212	6 weeks	25 and 50	Agomelatine at 25 mg was effective, but 50 mg may be beneficial for some patients without reducing tolerability
Lemoine *et al.*, 2007	126	Randomized, double-blind comparison with venlafaxine study	Venlafaxine 75–150 mg/day	334	6 weeks	25 and 50	Agomelatine showed similar antidepressant efficacy earlier and greater efficacy in improving subjective sleep as compared to venlafaxine
Olié & Kasper, 2007	125	Double-blind, flexible dose, parallel-group, placebo controlled study	Placebo	238	6 weeks	25 (with dose adjustment at two weeks to 50 mg/day in patients with insufficient improvement)	Agomelatine was significantly more efficacious than placebo. Agomelatine had a safety profile similar to placebo
Kennedy *et al.*, 2008	128	Randomized, double-blind comparison with venlafaxine study	Venlafaxine XR 150 mg/day	277	12 weeks	50	Agomelatine showed similar antidepressant efficacy with a superior sexual side effect profile to venlafaxine
Goodwin *et al.*, 2009	144	Randomized, double-blind, placebo-controlled study	Placebo	339	24 weeks	25 and 50	Agomelatine was more effective than placebo and prevented relapses without evidence of discontinuation symptoms
Hale *et al.*, 2010	130	Randomized, double-blind comparison with fluoxetine study on severely depressed patients (HAMD ≥ 25)	Fluoxetine 20–40 mg/day	515	8 weeks	25 and 50	Agomelatine was statistically more effective than fluoxetine
Kasper *et al.*, 2010	129	Randomized, double-blind comparison with sertraline study	Sertraline 50–100 mg/day	313	6 weeks	25 and 50	Agomelatine was more effective than sertraline. Agomelatine improved the circadian rest-activity cycle more than sertraline
Zajecka *et al.*, 2010	146	Multicenter, randomized, double-blind, placebo-controlled study	Placebo	511	8 weeks	25 and 50	Agomelatine at 50 mg showed greater and rapid reduction in all core symptoms of depression compared with placebo
Stahl *et al.*, 2010	147	Randomized, double-blind, placebo-controlled study	Placebo	503	8 weeks	25 and 50	Agomelatine at 25 mg was more effective than placebo over the course of the study, whereas agomelatine at 50 mg provided evidence for its antidepressant efficacy until week six, but not at study end
Quera-Salva *et al*., 2011	131	Randomized, double-blind comparison with escitalopram study	Escitalopram 10–20 mg/day	138	24 weeks	25 and 50	Agomelatine was as effective as escitalopram. Treatment with agomelatine improved morning condition and reduced daytime sleepiness compared with escitalopram
Martinotti *et al.* 2012	127	Open-label parallel-group, randomized comparison with venlafaxine study	Venlafaxine 75–150 mg/day	60	8 weeks	25 and 50	Agomelatine antidepressant efficacy proved to be similar to that of venlafaxine during an eight-week treatment period
Karaiskos *et al.* 2013	132	Observational open label, randomized, comparison with sertraline study	Sertraline 50–100 mg/day	40 depressed patients with non-optimally controlled type 2 diabetes mellitus (DM)	4 months	25 and 50	Agomelatine was effective in the treatment of depression and anxiety, as well as in the improvement of health-related behaviors, in depressed patients with non-optimally controlled type 2 DM
